# Supplementary material for: Empirical methods for controlling false positives and estimating confidence in ChIP-Seq peaks
Source: BMC Bioinformatics. 2008 Dec 5;9:523. doi: 10.1186/1471-2105-9-523 (PMC2628906; doi:10.1186/1471-2105-9-523)
Supplement: Additional File 1 — A variety of html documents from the USeq web site detailing the available applications, their best usage, output file type descriptions, command line menus, etc. [file 1471-2105-9-523-S1.zip › USeqUserGuides/JavaDoc.zip!/JavaDoc/trans/cel/SplineScalar.html]

SplineScalar


|  |  |  |
| --- | --- | --- |
| **Overview** \| **Package** \| **Class** \| **Use** \| **Tree** \| **Deprecated** \| **Index** \| **Help** | |  |
| **PREV CLASS**   **NEXT CLASS** | **FRAMES**    **NO FRAMES**     **All Classes** |  |
| SUMMARY: NESTED \| FIELD \| CONSTR \| METHOD | DETAIL: FIELD \| CONSTR \| METHOD |  |


---


## trans.cel Class SplineScalar

```
java.lang.Object
  trans.cel.SplineScalar
```

---

``` public class SplineScalar extends java.lang.Object ```

Generates multiplicative scalars based on a set of points using a combination of a cubic spline for values within the provided points
and linear regression for points outside the value range. Lagrange Interpolation might be something possibly more appropriate.
Assumes that each sucessive point is >= than last.

---

**Constructor Summary**

`SplineScalar(double[] modelPoints)`
  
          Assumes modelPoints are sorted min to max.


| **Method Summary** | |
| --- | --- |
| `LinearRegression` | `getLeftLR()` |
| `double[]` | `getModelPoints()` |
| `LinearRegression` | `getRightLR()` |
| `CubicSpline` | `getSpline()` |
| `static void` | `main(java.lang.String[] test)` |
| `double` | `scalar(double testValue, SplineScalar testScalar)`             Use to calculate a scaler that when multiplied by the testValue brings the testValue up to the model. |

**Methods inherited from class java.lang.Object**

`equals, getClass, hashCode, notify, notifyAll, toString, wait, wait, wait`

**Constructor Detail**

### SplineScalar

```
public SplineScalar(double[] modelPoints)
```

:   Assumes modelPoints are sorted min to max.

    **Parameters:**: `modelPoints` - - the points that represent the average/ model you want to generate scalars for.


**Method Detail**

### scalar

```
public double scalar(double testValue,
                     SplineScalar testScalar)
```

:   Use to calculate a scaler that when multiplied by the testValue brings the testValue up to the model.

    :   **Parameters:**: `testValue` - a non zero double **Returns:**: scaler x testValue = model.

---


### main

```
public static void main(java.lang.String[] test)
```

---


### getLeftLR

```
public LinearRegression getLeftLR()
```

---


### getModelPoints

```
public double[] getModelPoints()
```

---


### getRightLR

```
public LinearRegression getRightLR()
```

---


### getSpline

```
public CubicSpline getSpline()
```


---


|  |  |  |
| --- | --- | --- |
| **Overview** \| **Package** \| **Class** \| **Use** \| **Tree** \| **Deprecated** \| **Index** \| **Help** | |  |
| **PREV CLASS**   **NEXT CLASS** | **FRAMES**    **NO FRAMES**     **All Classes** |  |
| SUMMARY: NESTED \| FIELD \| CONSTR \| METHOD | DETAIL: FIELD \| CONSTR \| METHOD |  |


---
